# Longitudinal relationship of amino acids and indole metabolites with long-term body mass index and cardiometabolic risk markers in young individuals

**DOI:** 10.1038/s41598-020-63313-z

**Published:** 2020-04-14

**Authors:** Kolade Oluwagbemigun, Andrea Anesi, Maria Ulaszewska, Gerard Clarke, Ute Alexy, Matthias Schmid, Michael Roden, Christian Herder, Fulvio Mattivi, Ute Nöthlings

**Affiliations:** 1 Nutritional Epidemiology, Department of Nutrition and Food Sciences, University of Bonn, Bonn, Germany; 2 Department of Food Quality and Nutrition, Research and Innovation Centre, Fondazione Edmund Mach (FEM), San Michele all’Adige, Italy; 3 APC Microbiome Ireland, University College Cork, Cork, Ireland; 4 INFANT Research Centre, University College Cork, Cork, Ireland; 5 Department of Psychiatry and Neurobehavioural Science, University College Cork, Cork, Ireland; 6 Department of Medical Biometry, Informatics and Epidemiology, University Hospital Bonn, University of Bonn, Bonn, Germany; 7 Division of Endocrinology and Diabetology, Medical Faculty, Heinrich Heine University Düsseldorf, Düsseldorf, Germany; 8 Institute for Clinical Diabetology, German Diabetes Center, Leibniz Center for Diabetes Research at Heinrich Heine University Düsseldorf, Düsseldorf, Germany; 9 German Center for Diabetes Research (DZD), München-Neuherberg, Germany; 10 Department of Physics, University of Trento, San Michele all’Adige, Italy

**Keywords:** Predictive markers, Epidemiology

## Abstract

Amino acid metabolites in biofluids are associated with high body mass index (BMI) and cardiometabolic abnormalities. However, prospective investigations regarding these associations are few, particularly among young individuals. Moreover, little is presently known about the impact of long-term high BMI. Using data from the DOrtmund Nutritional and Anthropometric Longitudinally Designed study (111 males and 107 females), we prospectively investigated relations between repeatedly measured urinary levels of 33 metabolites and (1) previously identified long-term BMI trajectory groups from childhood into late adolescence and (2) cardiometabolic risk markers in late adolescence–young adulthood, in sex-specific linear mixed regression models. Males with long-term overweight had lower indole-3-acetic acid when compared to others. Further, methionine, isoleucine, tryptophan, xanthurenic acid, and indole-3-carboxaldehyde were negatively associated with C-reactive protein (CRP), but 5-hydroxyindole-3-acetic acid was positively associated with CRP. No associations were observed in females. Long-term overweight from childhood into late adolescence is associated with decreased urinary levels of gut bacteria-derived indole-3-acetic acid, and several urinary amino acids, including gut bacteria-derived indole-3-carboxaldehyde are associated with elevated CRP later on in life. Taken together, our data suggest that indole metabolites, and their gut bacteria producers play potentially important roles in overweight-related inflammation.

## Introduction

High body mass index (BMI) during childhood and adolescence is still a major public health concern^1,2^. The condition contributes to the development of cardiometabolic abnormalities such as inflammation over the life course^3^. Dysregulated metabolism is one of the biological mechanisms through which high BMI contributes to the development of these cardiometabolic abnormalities^4,5^. Scientific efforts have focused on carbohydrate and lipid metabolic perturbations as one of these mechanisms, interest in the impact of unfavorable alterations in protein/amino acid metabolism is now emerging^6,7^. Therefore, it would be necessary to explore the link of high BMI, amino acid metabolites, and cardiometabolic abnormalities.

Epidemiological studies in children and adolescents have reported positive association between BMI and biofluid amino acid and/or their metabolites such as alanine^8,9^, valine^8–10^, leucine^8,10,11^, isoleucine^8,10,11^, phenylalanine^8,11^, tyrosine^8,9,11^, histidine^8^, cysteine^12^, tryptophan-derived metabolites^13^, kynurenic acid^9,11^, kynurenine^11^, the kynurenine to tryptophan ratio (KTR, an index of increased metabolism of tryptophan along the kynurenine pathway)^14^, lysine, methionine, γ-glutamyltyrosine, asparagine, glycine, serine^11^, and branched-chain amino acids^15^. Conversely, negative association between BMI and leucine, isoleucine, phenylalanine, arginine, histidine, serine, and citrulline^4^, glutamine^9^, indole-3-propionic acid^16^, and 5-hydroxyindole acetic acid^17^ are also documented. Some of these findings are restricted to males^14,17^. These BMI-related changes in amino acid profiles might be a consequence of higher or lower intake of specific protein-containing foods^18^, changes in the activities of catabolizing host and/or bacterial enzymes^18,19^, changes in the redox status of the adipose tissue and liver^19^, and down-regulation of the expression of adipose tissue amino acid metabolizing enzymes and uptake of amino acids by adipose tissue^20^.

Similarly, amino acid metabolites are positively related to cardiometabolic risk markers (CRM) in children, adolescents, and young adults, albeit with a few exceptions. The positive associations include branched-chain amino acids (BCAAs) with insulin resistance (IR)^15,21,22^, triglycerides^23^, and triglycerides to high-density lipoprotein-cholesterol (HDL-C) ratio^24^; valine, leucine, and isoleucine^10^, phenylalanine and tyrosine^21^, and kynurenine and KTR^25^ with IR; cysteine with fasting plasma glucose (FPG), leptin, and IR;^12^ indole-3 propionic acid with HDL-C^16^; arginine, histidine, and serine with insulin sensitivity^5^; KTR with C-reactive protein (CRP), interferon-gamma, interleukin (IL)−6, IL-10, and alpha-1-acid glycoprotein^26^; and isoleucine and valine with IR^27^. The few reported negative associations include BCCAs with FPG^23^ and adiponectin^24^; tryptophan with CRP, interferon-gamma, IL-6, IL-10, and alpha-1-acid glycoprotein^26^; and numerous amino acids with CRP and IL-6^28^. The relation of BCCA with BCCAs with FPG^23^ and adiponectin^24^, and that of isoleucine and valine with IR^27^ are restricted to males, while the relation of BCAA with triglycerides^23^, and triglycerides to HDL-C ratio^24^ are restricted to females. The mechanistic involvement of BCCAs in the development of IR includes persistent activation of mTOR signaling pathway and accumulation of toxic BCAA metabolites that trigger mitochondrial dysfunction^29^, and the involvement of tryptophan and KTR in inflammation is through the immunoregulatory role of indoleamine 2,3-dioxygenase 1^30^.

Notably, only a few of the aforementioned studies are prospective^9,21,24–26^. Thus, more prospective investigations are needed. Moreover, the one-time measurement of BMI in earlier studies indicate a relation between a static state of body composition and amino acid metabolites. Given that body composition is dynamic, particularly among children and adolescents^31^, it would be necessary to investigate whether long-term BMI is associated with similar or different amino acid metabolites. Moreover, our knowledge of the relation between BMI and amino acid metabolites, and between amino acid metabolites and CRM might be incomplete because earlier studies sampled amino acids only at one time point. Static sampling of amino acids does not adequately capture the complex interplay of the several phases of host metabolism^19^. Further, some findings being restricted to either males or females warrants sex-specific analysis. Surely, aberrant amino acid metabolites may reveal metabolites and/or metabolic pathways perturbed as a result of a high BMI and identification of amino acids related to CRM may improve our understanding of the pathophysiological mechanisms underlying cardiometabolic abnormalities.

In a group of apparently healthy young individuals in whom BMI was measured annually over a 15-year period from childhood to late adolescence, we previously identified distinct long-term BMI trajectory groups^32^. In a subset of this study sample, we therefore sought in sex-specific analysis to (1) examine association between long-term BMI trajectory groups and repeatedly measured urine metabolites, mostly amino acids, during childhood and adolescence, and (2) investigate whether these metabolites are prospectively associated with the levels of 11 CRM in late adolescence–young adulthood.

## Results

### Study population

Our study population comprised 111 males and 107 females. As published before^32^, overweight trajectory was present only in males, and more males followed the high-normal weight trajectory as compared to females. Urine was sampled in majority of individuals at ages 9, 17, 18. In late adolescence–young adulthood, males had higher levels of SBP and FPG, while females had higher HDL-C, CRP, adiponectin, chemerin, and leptin. Males were heavier and longer than females at birth, males consumed more energy and protein daily as compared to females, and a lower percentage of mothers of males were employed as compared to mothers of females (Table 1).

**Table 1:** Basic characteristics of the study population, 111 males and 107 females.

	n (males, females)	Male	Female	*p-value*
**BMI trajectory groups**^**a**^				<0.001
Overweight	111, 107	16 (14)		
High-normal weight	111, 107	40 (36)	30 (28)	
Mid-normal weight	111, 107	33 (30)	40 (37)	
Low-normal weight	111, 107	22 (20)	37 (35)	
**Urine sampling at ages 9, 17, and 18**^**a**^	111, 107	57 (51)	64 (60)	0.201
**Cardiometabolic risk markers in late adolescence–young adulthood**^**b**^				
Systolic blood pressure, mmHg	111, 106	118 (110, 128)	110 (104, 118)	<0.001
Diastolic blood pressure, mmHg	111, 106	72 (66, 78)	70 (64, 76)	0.101
Serum triglyceride, mg/dL	106, 102	79 (60, 112)	89 (68, 113)	0.746
Serum HDL-C, mg/dL	108, 103	49 (41, 58)	65 (55, 73)	<0.001
Fasting plasma glucose, mg/dL	111, 105	95 (88, 100)	90 (86, 95)	<0.001
C-reactive protein, mg/dL	111, 106	0.05 (0.03, 0.14)	0.12 (0.06, 0.26)	<0.001
Interleukin-6, pg/mL	110, 106	0.7 (0.50, 1.04)	0.68 (0.48, 1.08)	0.827
Interleukin-18, pg/mL	109, 106	254.90 (210.51, 308.49)	247.92 (205.39, 306.55)	0.784
Adiponectin, ng/mL	110, 105	5910 (4389, 9241)	8758 (6512, 12976)	<0.001
Chemerin, ng/mL	111, 106	140.86 (122.46, 162,52)	166.99 (149.66, 189.12)	<0.001
Leptin, pg/mL	111, 102	2402 (1122, 5097)	12385 (8290, 19967)	<0.001
**Dietary intake and physical activity**				
Energy, kcal/day^b^	108, 102	2483 (2232, 2805)	1836 (1642, 2034)	<0.001
Protein, g/day^b^	108, 102	84.62 (75.14, 94.40)	59.21 (53.21, 67.71)	<0.001
Protein (%Energy)^a^	108, 102	13.52 (12.72, 14.57)	13.54 (12.20, 14.44)	0.347
Physical activity, metabolic equivalent task, hours/week^b^	105, 99	53.82 (30.00, 68.65)	33.75 (17.95, 47.71)	<0.001
**Prenatal, birth, and early life**				
Birth weight, g^b^	111, 107	3580 (3210, 3850)	3380 (3100, 3680)	<0.001
Birth length, cm^b^	111, 107	52 (51, 54)	51 (50, 53)	<0.001
Maternal BMI, kg/m2^b^	109, 107	22.94 (21.11, 26.09)	22.47 (20.59, 25.47)	0.387
Maternal gestational weight gain, kg^b^	108, 106	12 (10, 15)	12.5 (10, 15)	0.362
Breastfeeding duration, weeks^b^	111, 107	23 (8, 35)	26 (7, 34)	0.943
First-born child^a^	111, 107	65 (59)	65 (61)	0.264
Maternal education, high^a^	79, 96	75 (95)	87 (91)	0.826
Maternal employment, employed^a^	78, 96	14 (13)	34 (32)	<0.001
Smoking household^a^	82, 88	23 (28)	25 (28)	0.882

^a^n(%); ^b^Median (25^th^ percentile, 75th percentile). n = count, % = percentage, BMI = Body mass index, HDL-C = High-density lipoprotein-cholesterol.

Further details of the study participants’ age at urine sampling (metabolite measurement) are shown in Suppl. Table 1a,b. In both sexes, over half of the participants had their three measurements at ages 9, 17, and 18, and about 10% had measurements at ages 9, 16, and 17.

### Multivariable linear regression

#### The association between BMI trajectory and metabolites

As shown in Suppl. Table 2, except for kynurenine and KTR in males, the linear time models yielded generally a better fit for the metabolites. Therefore, except for these two metabolites which we fitted with quadratic time models, all the remaining metabolites in both sexes were fitted with linear time models.

Table 2 shows the *p*-values and *q*-values of the association between BMI trajectory group and each of the 32 metabolites and KTR in males and females. The results indicate that at *p*-value ≤ 0.05 and *q*-value ≤ 0.05, BMI trajectory group was significantly associated with 3-methoxy-p-tyramine (*p*-value = 0.005, *q*-value = 0.045), indole-3-acetamide (*p*-value = 0.005, *q*-value = 0.045), and indole-3-acetic acid (*p*-value = 0.0001, *q*-value = 0.002) in males. However, BMI trajectory group was not associated with any metabolites in females. For this reason, subsequent mean comparisons were performed for only 3-methoxy-p-tyramine, indole-3-acetamide, and indole-3-acetic acid in males.

**Table 2:** The *p*-values and *q*-values of the association of body mass index trajectory group with 32 metabolites and kynurenine to tryptophan ratio in males and females.

				**MALES**					
	GABA	Met	Val	Leu	Ile	PA	QA	5-HT	DA
*p*-value	0.886	0.032	0.179	0.222	0.107	0.062	0.804	0.010	0.153
*q*-value	0.886	0.103	0.304	0.355	0.214	0.151	0.854	0.069	0.283
	Tyr	Phe	Tyra	**3-Me-Tyra**	HVA	KA	5-OH-IAA	Trp	
*p*-value	0.466	0.524	0.036	**0.005**	0.072	0.026	0.042	0.252	
*q*-value	0.566	0.614	0.103	**0.045**	0.163	0.103	0.110	0.372	
	Kyn	XA	**IACT**	**IAA**	ILA	IPA	IALD	ICA	
*p*-value	0.096	0.036	**0.005**	**0.0001**	0.025	0.031	0.833	0.343	
*q*-value	0.204	0.103	**0.045**	**0.002**	0.103	0.103	0.859	0.485	
	AA	3-OH-AA	Try	5-Me-IAA	TrpME	5-OH-Trp	3-OH-kyn	KTR	
*p*-value	0.781	0.617	0.370	0.229	0.015	0.434	0.158	0.383	
*q*-value	0.854	0.699	0.501	0.355	0.083	0.546	0.283	0.501	
				**FEMALES**					
	GABA	Met	Val	Leu	Ile	PA	QA	5-HT	DA
*p*-value	0.439	0.812	0.485	0.131	0.258	0.843	0.940	0.888	0.966
*q*-value	0.755	0.966	0.755	0.755	0.755	0.966	0.966	0.966	0.966
	Tyr	Phe	Tyra	3-Me-Tyra	HVA	KA	5-OH-IAA	Trp	
*p*-value	0.256	0.483	0.028	0.367	0.322	0.317	0.511	0.493	
*q*-value	0.755	0.755	0.471	0.755	0.755	0.755	0.755	0.755	
	Kyn	XA	IACT	IAA	ILA	IPA	IALD	ICA	
*p*-value	0.787	0.964	0.024	0.158	0.949	0.885	0.480	0.121	
*q*-value	0.966	0.966	0.471	0.755	0.966	0.966	0.755	0.755	
	AA	3-OH-AA	Try	5-Me-IAA	TrpME	5-OH-Trp	3-OH-kyn	KTR	
*p*-value	0.440	0.216	0.076	0.301	0.924	0.450	0.737	0.388	
*q*-value	0.755	0.755	0.755	0.755	0.966	0.755	0.966	0.755	

GABA = Gamma-aminobutyric acid; Met = Methionine; Val=Valine; Leu = Leucine; Ile=Isoleucine; PA = Picolinic acid; QA = Quinolinic acid; 5-HT = Serotonin; DA = Dopamine; Tyr = Tyrosine; Phe=Phenylalanine; Tyra = Tyramine; 3-Me-Tyra = 3-methoxy-p-tyramine; HVA = Homovanillic acid; KA = Kynurenic acid; 5-OH-IAA = 5-hydroxyindole-3-acetic acid; Trp = Tryptophan; Kyn=Kynurenine; XA = Xanthurenic acid; IACT = Indole-3-acetamide; IAA = Indole-3-acetic acid; ILA = Indole-3-lactic acid; IPA = Indole-3-propionic acid; IALD = Indole-3-carboxaldehyde; ICA = Indole-3-carboxylic acid; AA = Anthranilic acid; 3-OH-AA = 3-hydroxyanthranilic acid; Try = Tryptamine; 5-Me-IAA = 5-Methoxyindole-acetic acid; TrpME = Tryptophan methyl ester; 5-OH-Trp = 5-hydroxy-tryptophan, 3-OH-Kyn = 3-hydroxykynurenine. KTR = Kynurenine to Tryptophan ratio

The bolded metabolites indicate significant association with BMI trajectory at *p*-value ≤ 0.05 and *q*-value ≤ 0.05

*Metabolites and time were normalized before statistical analyses.

**Figure 1:**
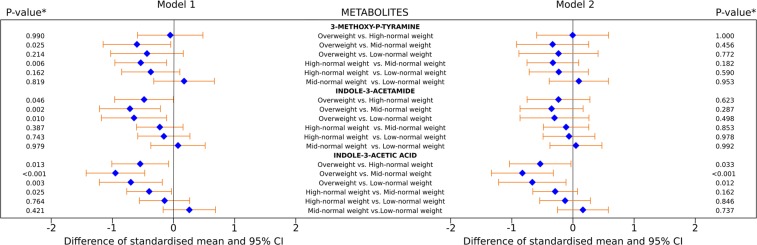
Mean difference in standard-deviation scores of 3-methoxy-p-tyramine, indole-3-acetamide, and indole-3-acetic acid between pairs of body mass index trajectory groups in males. Model 1: left, Model 2: right. Model 1: adjusted for time. Model 2: adjusted for time, birth weight and length, maternal BMI, maternal pregnancy weight gain, breastfeeding duration, birth order, maternal education, and maternal employment, smoking in household, physical activity, daily energy intake, percentage of energy from protein, and two-way interactions of physical activity, daily energy intake, and percentage of energy from protein with BMI trajectory. *Simulated adjusted *p*-value.

The results of the marginal mean difference in standard-deviation (SD) scores of 3-methoxy-*p*-tyramine, indole-3-acetamide, and indole-3-acetic acid between the BMI trajectory groups in males are shown in Fig. 1. Adjusting for time (model 1), the overweight and high-normal weight trajectory groups had lower 3-methoxy-p-tyramine as compared to the mid-normal weight trajectory group. The overweight trajectory had lower indole-3-acetamide and indole-3-acetic acid as compared to all remaining three trajectory groups. Additionally, the high-normal weight trajectory group had lower indole-3-acetic acid as compared to the mid-normal weight trajectory group (Fig. 1, left). After full covariate adjustment (model 2), the overweight trajectory had 0.54 SD, 0.83 SD, 0.67 SD lower indole-3-acetic acid as compared to the high-normal weight (−0.54: −1.04; −0.033, adjusted *p-value* =  0.033), mid-normal weight (−0.83: −1.33; −0.32, adjusted *p-value* =   < 0.001), and low-normal weight (−0.67: −1.22; −0.11, adjusted *p-value* =  0.012) trajectory groups, respectively (Fig. 1, right).

In humans, ultimate source of indole-3-acetic acid is tryptophan. Hence, in sensitivity analyses, BMI trajectory group differences in indole-3-acetic acid were further adjusted for tryptophan. Adjusting for this precursor slightly attenuated the above result (Suppl. Table 3). This result was similar with further adjustment for urine creatinine or osmolality.

### The association between metabolites and kynurenine and tryptophan ratio, and cardiometabolic risk markers

Collectively, the metabolites and KTR explained between 26–54% and 10–89% of the variance in CRM in males and females, respectively (Table 3). However, at *p*-value ≤ 0.05 and *q*-value ≤ 0.05 only the explained variance of CRP in males was statistically significant. The metabolites and KTR collectively explained 54% of the variance in CRP in males (*p*-value = 0.002, *q*-value = 0.025). Therefore for subsequent analysis, we reported associations between these metabolites and CRP in males.

**Table 3:** Overall significance of regression model with cardiometabolic risk markers as dependent variable and 32 metabolites and kynurenine and tryptophan ratio as independent variables.

Cardiometabolic risk markers*	MALES			FEMALES		
	*p*-value	*q*-value	R-square values	*p*-value	*q*-value	R-square values
Systolic blood pressure	0.811	0.893	0.28	0.716	0.868	0.71
Diastolic blood pressure,	0.017	0.095	0.49	0.218	0.560	0.22
Serum triglyceride	0.298	0.468	0.37	0.314	0.576	0.31
Serum high-density lipoprotein-cholesterol	0.122	0.384	0.41	0.891	0.891	0.89
Fasting plasma glucose	0.230	0.422	0.39	0.244	0.560	0.24
**C-reactive protein**	**0.002**	**0.025**	**0.54**	0.485	0.761	0.48
Interleukin-6	0.448	0.616	0.35	0.149	0.560	0.15
Interleukin-18	0.175	0.384	0.40	0.567	0.779	0.57
Adiponectin	0.164	0.384	0.41	0.789	0.868	0.79
Chemerin	0.676	0.8268	0.31	0.096	0.560	0.10
Leptin	0.898	0.898	0.26	0.254	0.560	0.25

The bolded cardiometabolic risk marker indicate a significant model at p-value ≤ 0.05 and q-value ≤ 0.05

*Metabolites and cardiometabolic risk markers were normalized before statistical analysis.

**Figure 2:**
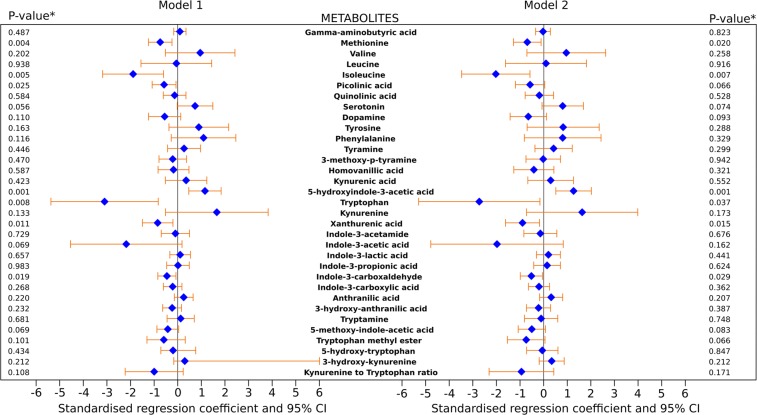
Association of 32 metabolites and kynurenine to tryptophan ratio with CRP in males. Model 1: left, Model 2: right. Model 1 comprises all 32 metabolites and kynurenine and tryptophan ratio. Model 2 was Model 1 adjusted for birth weight and length, maternal BMI, maternal pregnancy weight gain, breastfeeding duration, birth order, maternal education, and maternal employment, smoking in household, physical activity, daily energy intake, percentage of energy from protein, and BMI trajectory. *Multivariable adjusted *p*-value.

As shown in the model comprising only the metabolites (model 1), methionine, isoleucine, picolinic acid, tryptophan, xanthurenic acid, and indole-3-carboxaldehyde were negatively associated with CRP, but 5-hydroxyindole-3-acetic acid was positively associated with CRP (Fig. 2, left). Following full covariate adjustment (model 2), a 1 SD increase in methionine (β = −0.70: −1.29; −0.12, *p-value* = 0.02), isoleucine (β = −2.03: −3.48; −0.59, *p-value* = 0.007), tryptophan (β = −2.74: −5.30; −0.17, p-value = 0.037), xanthurenic acid (β = 1 = −0.90: −1.62; −0.18, *p-value* = 0.015), and indole-3-carboxaldehyde (β = −0.53: −1.00; −0.06, *p-value* = 0.029) were associated with a 0.7 SD, 2 SD, 2.7 SD, 0.9 SD, and 0.5 SD decrease in CRP, respectively. Further, a 1 SD increase in 5-hydroxyindole-3-acetic acid (β = 1.26: 0.51; 2.01, *p-value* = 0.001) was associated with a 1.3 SD increase in CRP (Fig. 2, right). Indole-3-carboxaldehyde has the narrowest 95% CI, therefore its significant association with CRP has the least uncertainty. These results were essentially unaltered following additional adjustment for urine creatinine or osmolality.

## Discussion

The aims of this study were to examine sex-specific associations between long-term BMI trajectory groups and repeatedly measured urine metabolites, mostly amino acids, among apparently healthy young individuals, and to investigate whether these metabolites are associated with the levels of subsequent CRM in late adolescence–young adulthood. We observed that individuals who belonged to the overweight trajectory group, that is, persistently overweight from childhood into late adolescent had lower levels of urinary indole-3-acetic acid when compared to other individuals. Furthermore, we found that methionine, isoleucine, tryptophan, xanthurenic acid, and indole-3-carboxaldehyde levels were independently negatively, and 5-hydroxyindole-3-acetic acid levels were independently positively associated with CRP in late adolescence–young adulthood. Interestingly, both findings were restricted to males.

Our sex-specific findings extends previous findings of studies in children and adolescents comprising both sexes. This includes a study reporting that high BMI was associated with a decreased KTR in males, but not in females^14^. Moreover, BCCAs were associated with a decreased FPG in males, but increased triglycerides in females^23^ as well as with a decreased adiponectin in males, but with increased triglycerides to HDL-C ratio in females^24^. The fact these studies^14,23,24^ and ours consistently showed inverse associations in males suggest that our findings are unlikely to be spurious. Possible explanations for these observed sex-specific findings include sex-related differences in protein turnover, amino acid handling systems, and the influence of sex hormones^33^. Sex-related differences are thought to primarily develop after adolescence due to the more marked hormonal differences^33^. However, considering that our study included measurement of metabolites in childhood, either indicate that an impact of sex hormones occur before adolescence, or that all the aforementioned mechanisms provide a complete explanation of the sex-specific findings. These sex-specific findings warrant further investigation.

Overweight, either cross-sectionally or prospectively has not been previously linked to indole-3-acetic acid in children and adolescents. The few cross-sectional studies in adults regarding indole-3-acetic acid have yielded mixed results^34–37^. In agreement with our study, Liu *et al*. observed that BMI negatively correlated with sputum indole-3-acetic acid^35^, a result at variance with others in which no relation was observed between BMI and serum indole-3-acetic acid^34^, between BMI and faecal indole-3-acetic acid^36^, and between BMI and plasma indole-3-acetic acid in older men^37^. Clearly, BMI trajectory group differences being observed for only indolic tryptophan metabolite indicate that the impact of long-term BMI is very specific and does not result in a global disruption of all amino acid metabolism. However, our study failed to demonstrate an association with indole-3-propionic acid, an indole metabolite that has been cross-sectionally related to BMI in children and adolescents^16^. Indeed, the consistent inverse relation of BMI with the indole metabolites in our study and Farook *et al*.^16^ suggest that high BMI impact indolic tryptophan metabolism, with the resulting metabolites being dependent on the duration of high BMI. Indole-3-acetic acid have been shown to drive the emergence of obesity by acting on the extended reward network^36^, however the current study modeled the effect of BMI trajectory group since it makes intuitive sense that changes in BMI impacted the levels of indole-3-acetic acid and our BMI measurements precedes metabolite measurements. Nonetheless, one cannot exclude the presence of a bidirectional association between BMI and indole-3-acetic.

In humans, indole-3-acetic acid is a tryptophan metabolite that are largely generated by the direct or indirect metabolism of the gut microbiota^38,39^. Approximately 4–6% of tryptophan undergoes bacterial degradation to yield indole metabolites^40^. Since human de novo production of indole metabolites is unlikely^40^, the levels of indole-3-acetic acid is either a reflection of dietary intake (in this case, tryptophan-containing foods and cruciferous vegetables) that are modulated by the gut bacteria or entirely a reflection of changes in gut microbial metabolism. There is a correlation between vegetable intake and calorie intake^41^, therefore controlling for calorie intake and protein intake, and their interaction with BMI trajectory group minimizes the influence of differential consumption of cruciferous vegetables and tryptophan-containing foods on our findings. The fact that tryptophan only minimally attenuate BMI trajectory group differences in indole-3-acetic acid also suggests that this association is independent of this precursor.

*Clostridium bartlettii*, several *Bacteroides* species and other bacteria have been reported to produce indole-3-acetic acid^42^. This suggests that in individuals with long-term overweight, dysbiosis and/or altered metabolic functions of the gut microbiota results in decreased synthesis or increased utilization of indole-3-acetic acid. Although the potential of the gut bacteria of healthy humans to produce indole is very heterogeneous;^43^ the absence^44^ and lower levels^45^ of indoles in serum of germ-free animals compared to their conventionally raised counterparts suggest that these bacteria will be either absent or decreased in overweight individuals. Prospectively linking long-term overweight status with the abundance and function of the gut microbiota would help identify the altered bacteria, and linking the identified bacteria to levels of indole-3-acetic acid would help identify its producer in our study population. There are other gut microbiota-controlled tryptophan pathways^46^. Therefore, whether and the extent to which long-term overweight affects these pathways should be evaluated in future studies. Ultimately, indole-3-acetic acid may help to further characterize young individuals with different long-term BMI phenotypes. Overall, indolic tryptophan metabolic pathway seems to be highly dysregulated by long-term overweight.

Secondly, we observed that the levels of methionine, isoleucine, tryptophan, xanthurenic acid, and indole-3-carboxaldehyde from childhood into late adolescent were inversely related to CRP levels in late adolescence–young adulthood, but the levels of 5-hydroxyindole-3-acetic acid was directly related to CRP–a well-established marker of inflammation. Interestingly, all these metabolites are independently related to CRP, indicating that their association is not confounded by their precursors. While there is some evidence that changes in amino acids occur in inflammatory states^47^, it is less clear if these changes are a cause or a consequence of inflammation. Only a few prospective investigations in children and adolescent have implicated amino acid metabolites in cardiometabolic risks^21,24–26^, and of these studies only one reported relations with CRP^26^. In agreement with our results, Kosek *et al*.^26^ found an inverse association between tryptophan and CRP. Similarly, a cross-sectional investigation, including children and adolescents, is in line with our findings of the inverse relation of methionine and tryptophan with CRP^28^.

To our knowledge, no previous study in childhood, adolescents, or young adults have prospectively linked methionine, isoleucine, xanthurenic acid, indole-3-carboxaldehyde, and 5-hydroxyindole-3-acetic acid to CRP. Nevertheless, cross-sectional investigations in older adults are in line with our findings. These include an inverse association of tryptophan^48^ and xanthurenic acid with CRP^49^, and a direct association between 5-hydroxyindole-3-acetic acid and CRP^50^. Other studies in adults that contrast our findings include lack of association of isoleucine^35^, lack of association of methionine and tryptophan^50^, and positive relation of isoleucine^51,52^ with CRP. Although no study have reported an association between indole-3-carboxaldehyde and CRP; exogenous administration of indole-3-carboxaldehyde decreased the production of several inflammatory cytokines^53^. Consistent with our findings of several amino acids being related to CRP, a review concluded that several amino acids are involved in the development of future cardiometabolic abnormalities such as IR^54^. Since.we adjusted for the precursors (tryptophan and serotonin) of indole-3-carboxaldehyde, xanthurenic acid, and 5-hydroxyindole-3-acetic acid, it is unlikely that their association with CRP is due levels of these precursors or intake of diets rich in them. Systemic inflammation drives amino acid metabolism^46^; however our longitudinal study provides a direct evidence that variation in amino acid metabolites predates systemic inflammation.

The inflammatory role of the CRP-related amino acid metabolites in the current study have been documented. Methionine, an aliphatic, sulfur-containing, essential amino acid exerts anti-inflammatory activity through the activation of endogenous antioxidant enzymes such as methionine sulfoxide reductase A, and by counteracting oxidative stress through the biosynthesis of glutathione^55^. Isoleucine, a branched chain amino acid exerts anti-inflammatory activity through interference with the action and/or synthesis of prostaglandins^56^, and inducing the expression of β-defensins via G-protein-coupling receptors and ERK/MAPK signaling pathways^57^. Tryptophan, an essential aromatic amino acid, plays a critical role in controlling systemic inflammatory responses through its endothelium-derived metabolite 5-methoxytryptophan^58^. Xanthurenic acid is one of the several metabolites in the kynurenine pathway of tryptophan metabolism^59^. It reduces interferon-gamma production^60^ and possesses powerful antioxidant properties^61^. Indole-3-carboxaldehyde (indole-3-aldehyde), a gut microbiota-derived indole derivative of tryptophan catabolism produced by the action of *Lactobacillus*-encoded tryptophanase inhibits inflammation through activation of the aryl hydrocarbon receptor in lymphoid cells^62^. Finally, 5-hydroxyindole-3-acetic acid is the most abundant end product of both central and peripheral enzymatic degradation of serotonin^63^. Although the direct biological activity of 5-hydroxyindole-3-acetic acid is yet to be documented; its excessive production by endocrine and neuroendocrine tumors^63^ and the large quantities of reactive oxygen species that are produced during its biosynthesis from serotonin^64^ might explain its association with systemic inflammation. Epidemiological studies have reported associations between proinflammatory state among adolescents and young adults and an increased risk of future cardiovascular diseases^65^, thus the amino acids associated with elevated CRP in the present study could be biomarkers of proinflammatory phase leading to the development cardiovascular diseases.

One major strength of this work is that it is a prospective study in apparently healthy young individuals. This prospective investigation indicates that the obtained exposure-outcome associations are reliable. Moreover, the repeated measurement of metabolites ensures that the metabolite levels of our participants are well captured, thereby providing greater confidence in our findings. In addition, for both research aims, we adjusted for protein and calorie intake since the metabolic fate of amino acids is dependent on their dietary availability. We also corrected for variations in physical activity and several early life factors. Only a few previous studies adequately controlled for these covariates. Finally, metabolites were profiled in urine which is a non-invasive biosample. Considering that the concentrations of several urine and plasma amino acids are correlated^66^, urine may be a substitute for plasma in profiling amino acids, especially in large epidemiological studies.

Notwithstanding the strengths of our study, one limitation is that this study is observational, so it cannot confirm causal relationships. It would be necessary to determine whether a causal relation exists between long-term BMI trajectory groups and these metabolites, and between these metabolites and CRP, and if it exists the magnitude of their causal relations. One approach that may improve causal inference of association of these metabolites with CRP within an observational study setting is by examining whether genetically determined levels of these metabolites are related to CRP in Mendelian randomization analysis. Secondly, the homogeneous nature of our study population, in terms of geographical location and socioeconomic background, may limit generalizability of our findings. These findings should be externally validated on more geographically, socioeconomically, and ethnically diverse populations. Besides, residual and unmeasured confounders such as medications might have influenced our results. Additionally, single imputation of covariates may have underestimated the uncertainty around the effect estimates in our adjusted models. Several host factors such as protein/amino acid absorption, catabolism and uptake, and excretion as well as kidney function might also confound our results. Future studies should consider the assessment of the aforementioned host factors such as measurement of faecal levels of these metabolites in order to rule out malabsorption. Moreover, our findings speculate about the role of the gut microbiota, without measuring the faecal levels of these metabolites. Faecal as well as circulating levels of these metabolites would have strengthened our findings.

In conclusion, this prospective investigation in apparently healthy young individuals shows that independent of several factors, males who were overweight from childhood into late adolescent have decreased urinary levels of gut bacteria-derived indole-3-acetic acid, and that several urinary amino acids, including gut bacteria-derived indole-3-carboxaldehyde, negatively predict serum CRP in late adolescence–young adulthood. Although exactly the same metabolite is not related to both long-term overweight and CRP, the fact that decreasing levels of indole metabolites-indole-3-acetic acid and indole-3-carboxaldehyde are associated with long-term overweight and elevated CRP, respectively suggests that indole metabolites and their gut bacteria producers play an imperative role in overweight-related inflammation among young individuals. These current findings further underline how the host phenotype and the microbiota interact to influence health outcomes. More human prospective studies and animal studies are needed to better understand the role of altered indolic tryptophan metabolism and the gut microbiota in overweight-associated systemic inflammation.

## Subjects and Methods

### Study design and participants

The study sample was selected from participants of the DOrtmund Nutritional and Anthropometric Longitudinally Designed (DONALD) study, an ongoing, open cohort study conducted in Dortmund, Germany. This study collects data on diet, growth, development and metabolism of healthy children and adolescents since 1985. In the first few study years, approximately 300 participants >2 years old were also recruited. Since then, approximately 35–40 infants are newly recruited every year. The regular visits begin at 3 months of age. The participants return for three more visits in the first year, two in the second year and thereafter annually until young adulthood. Yearly examinations include 3-day weighed dietary records, anthropometric measurements, collection of 24-h urine samples, interviews on lifestyle and medical examinations. Since 2005, participants >18 y are invited for subsequent examinations with fasting blood withdrawal. Parental examinations (anthropometric measurements, lifestyle interviews) take place every four years. Due to the specific design of the DONALD study, participants from well-educated mothers are overrepresented. Further details of the DONALD study were described elsewhere^67^. The study was approved by the Ethics Committee of the University of Bonn (approval number 098/06) and conducted according to the guidelines of the Declaration of Helsinki.

### Statement attesting to informed consent for study participation

For all examinations in the DONALD study, written consent was obtained from parent and/or legal guardian of participants during their childhood phase and written consent was also obtained from study participants themselves later on in life. A statement attesting to informed consent from a parent and/or legal guardian for study participation was provide as thus, “I consent to my child being medically examined and physically measured by qualified personnel. I am prepared to answer questions about my child regarding pregnancy and birth, development and diseases, as well as lifestyle factors and diet, and to prepare dietary protocols. I agree to answer questions about my own health status and lifestyle and to have my height and weight measured”.

### Study sample

**Figure 3:**
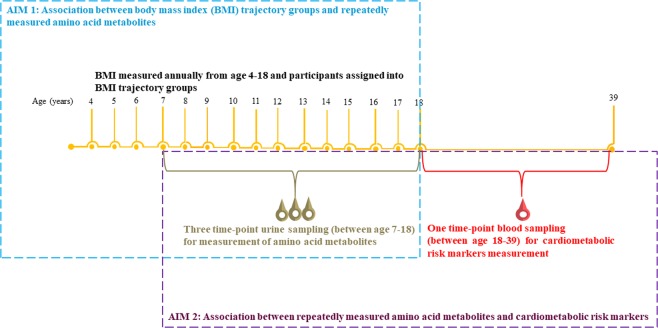
An overview of the study and its research aims.

The sample for the current analyses was a subset of 354 males and 335 females DONALD study participants from whom we previously identified long-term BMI trajectory groups from age four to 18^32^. These individuals are singletons, full term births (36 to 42 weeks), had a birth weight greater than or equal to 2500 g, and had at least one BMI measurement in childhood (4–9.9 years), early adolescence (10–14.9 years), and late adolescence (15–18 years). Four trajectory groups (overweight, high-normal weight, mid-normal weight, and low-normal-weight), and three trajectory groups (high-normal weight, mid-normal weight, and low-normal-weight) were identified in males and females, respectively^32^. Of these individuals, 218 (111 males and 107 females) individuals provided 24-hour urine samples at three time-points over the course of BMI measurement, and one blood sample in late adolescence–young adulthood (between age 18 and 39 years) (Fig. 3). The current analyses were based on data from these 111 males and 107 females.

Assessment of anthropometric variables, dietary intake and physical activity, early life and socio-economic characteristics

Anthropometric data for all participants were obtained by measurement that adhere to standard procedures during their annual study visit. BMI, standardized BMI z-scores, and their trajectories were determined, as previously reported^32^. Three-day weighed dietary records were used to collect nutritional data for three consecutive days. Using all three-day dietary data, the nutrient intake was computed using the continuously updated in-house nutrient database, LEBTAB, and the individual mean daily intake of energy and protein over the three record days was calculated. Further, physical activity was assessed using a detailed questionnaire about the duration and frequency of both organized and non-organized physical activity. Physical activity was then expressed as daily energy expenditure in metabolic equivalent task-hours. Parents were interviewed about familial and socio-economic characteristics. Information on birth anthropometrics were extracted from a standardized document (“Mutterpass”) given to all pregnant women in Germany. The BMI of the mother of the participants at study entry was also documented.

### Urine sampling, preparation, and metabolic profiling of metabolites

#### Urine sampling

Annual 24-h urine sampling were scheduled for participants older than 3 or 4 years. The sampling conducted at home follows a standardized procedure after detailed instruction to the families. Participants were asked to void their bladders upon getting up in the morning; this urine sample is completely discarded. This sets the start of the collection and which ends with voiding the bladder in the next morning. The participants store the urine sample in preservative-free, Extran-cleaned (Extran, MA03; Merck, Darmstadt, Germany) 1 L plastic containers at less than −12 °C. The samples were transferred to the study centre where they were stored at −22 °C until analysis. Details of urine sampling have been presented elsewhere^68^.

### Urine sample preparation and targeted metabolic profiling of metabolites

For each of the three 24-hour urine samples of the 111 males and 107 females in the current study, 25 µL aliquot and 225 µL of internal standard mix with water: acetonitrile 8:2 (v/v) were added (trypthophan-d5 at final concentration 1 ppm; tyrosine-d4, methionine-d4, serotonin-d4, kynurenic acid-d5, 5-hydroxyindole-acetic acid- d5 and dopamine d5 at 0.5 ppm), were loaded in 96 well multifilter plates (Millipore). The target metabolites were detected and quantified on a UHPLC-ESI-Triple-quadrupole-MS (Waters®), using an injection volume of 2 µL. Data processing was performed with Mass Lynx 4.1 software (Waters®). The 33 metabolites quantified were gamma-aminobutyric acid, methionine, valine, leucine, isoleucine, picolinic acid, quinolinic acid, serotonin, dopamine, tyrosine, phenylalanine, tyramine, 3-methoxy-p-tyramine, homovanillic acid, kynurenic acid, 5-hydroxyindole-3-acetic acid, tryptophan, kynurenine, xanthurenic acid, indole-3-acetamide, indole-3-acetic acid, indole-3-lactic acid, indole-3-propionic acid, indole-3-carboxaldehyde, indole-3-carboxylic acid, anthranilic acid, 3-hydroxyanthranilic acid, tryptamine, 5-methoxyindole-acetic acid, tryptophan methyl ester, 5-hydroxy-tryptophan, 3-hydroxykynurenine. The metabolites show a coefficient of variation lower than 1% and matrix effects, evaluated by the matrix match calibration approach, were minimal. Details of urine sample preparation and targeted metabolic profiling of amino acid metabolites can be found elsewhere^69^.

### Blood sampling and measurement of cardiometabolic risk markers

In late adolescence–young adulthood, venous blood samples were drawn after an overnight fast, centrifuged within 15 min, and frozen at −80 °C. Circulating concentrations of HDL-C, FPG, CRP, IL-6, IL-18, adiponectin, chemerin, and leptin were measured. HDL-C was measured with the Advia 1650-Chemistry System analyser (Siemens Healthcare Diagnostics, Eschborn, Germany, FPG on a Roche/Hitachi Cobas c 311 analyzer (Basel, Switzerland), triglycerides and CRP with the Roche/Hitachi Cobas c311 analyser (Roche diagnostics, Mannheim, Germany), IL-6 with the Human IL-6 Quantikine HS ELISA (R&D Systems, Wiesbaden, Germany), IL-18 with the Human IL‐18 ELISA (Medical and Biological Laboratories, Nagoya, Japan), adiponectin with the Human Total Adiponectin/Acrp30 Quantikine ELISA kit (R&D Systems), chemerin with the Human Chemerin ELISA (BioVendor, Brno, Czech Republic), and leptin with Leptin Quantikine ELISA (R&D System) as previously described^70,71^.

Systolic (SBP) and diastolic blood pressures (DBP) were measured by the standard procedure at the study center, according to standardized procedures with an automated oscillometric device (Datascope Accutorr Plus, Mahwah, NJ, USA). For each participant, three consecutive BP measurements were taken at 3-min intervals after an initial 5-min rest and following a non-strenuous part of the examination. During the measurements, the participant’s back was supported in an upright position, with the right forearm resting on a table at heart level, and the legs uncrossed with feet on the floor. Therefore, in the present study, eleven CRM-SBP and DBP, serum triglycerides, HDL-C, FPG, CRP, IL-6, IL-18, adiponectin, chemerin, and leptin were analysed.

### Statistical analyses

#### Participant characteristics

All statistical analyses were conducted using SAS 9.4 (SAS®, SAS Institute, Cary, NC). Sex-specific baseline characteristics of the study sample (n = 218, males = 111, females = 107) are presented as medians and corresponding 25th and 75th percentiles or as counts (percentages) where appropriate. Sex-specific differences in baseline characteristics according to sex were explored using the Mann–Whitney U test and chi square tests for continuous and categorical data, respectively.

### Multivariable linear regression

#### The association between BMI trajectory groups and metabolites

All 32 metabolites and the KTR were normalized by calculating their rank normal scores using the Blom method. We used linear mixed models (PROC MIXED) to examine the association between BMI trajectory (categorical predictor variable) and each of the 33 (32 metabolites and KTR) dependent variables.

First, we determined the trends for each of the dependent variables over time (participants’ ages) by fitting unconditional linear and quadratic time models. We examined the convergence information and selected a better fitting model between the linear and quadratic time models as the one which minimizes the corrected Akaike’s Information Criterion. Time was expressed as a deviation from its mean in order to reduce multicollinearity between the linear and quadratic terms of the curvilinear model when the quadratic model indicated a better fit. The REPEATED statement was included in the PROC MIXED, and the individual was specified as the subject. The Kenward-Roger estimation method was used for calculating degrees of freedom. Due to the unequally spaced repeated measurements, we specified an exponential spatial covariance structure.

Then, we included the BMI trajectory group (categorical) variable as fixed effect and tested its association with each dependent variable. We obtained the overall significance (type 3 F-tests p-value) of the BMI trajectory and estimated its corresponding false discovery rate (FDR) p-value (q-value) by PROC MULTTEST. A combined p-value ≤ 0.05 and q-value ≤ 0.05 corrected with respect to the 33 dependent variables, were considered statistically significant. Thus, model 1 includes BMI trajectory, time and BMI trajectory by time interaction (for linear model), with quadratic time, and BMI trajectory by quadratic time interaction (for curvilinear model).

When the BMI trajectory group effect was significant for a dependent variable in model 1, we further examined the heterogeneity across BMI trajectory groups as the marginal mean difference (and 95% Confidence interval, CI) in the dependent variable using the LSMEANS statement. The confidence limits of the mean differences and p-value were adjusted for multiple comparisons with the simulate method. Model 1 was further adjusted for directed acyclic graph-identified minimal adjustment sets of covariates of the association between BMI trajectory and the dependent variables (model 2). These covariates were birth weight and length, maternal BMI, maternal pregnancy weight gain, breastfeeding duration, birth order, maternal education, and maternal employment, smoking in household, physical activity, daily energy intake, percentage of energy from protein, and two-way interactions of physical activity, daily energy intake, and percentage of energy from protein with BMI trajectory.

### Association between metabolites and cardiometabolic risk markers

To address the second aim, that is, whether metabolites are related to the 11 CRM (SBP, DBP, triglycerides, HDL-C, FPG, CRP, IL-6, IL-18, adiponectin, chemerin, and leptin), we constructed a two-stage model. In the first stage, we modeled the normalized concentration of the 32 metabolites and KTR as a function of time using linear mixed models (PROC MIXED) with random coefficients and unstructured covariance-structure, and obtained individual-specific predicted value (best linear unbiased predictor, BLUP). In the second stage, in a multivariate multivariable linear model, the normalized CRM (dependent variables) were regressed on the BLUP estimates of the 33 independent variables (32 metabolites and KTR). We considered the CRM for which this model (model 1) showed significance at an overall F statistic p-value ≤ 0.05 and q-value ≤ 0.05. For significant model(s), metabolites were considered significant at p-value ≤ 0.05. In model 2, we adjusted for birth weight and length, maternal BMI, maternal pregnancy weight gain, breastfeeding duration, birth order, maternal education, and maternal employment, smoking in household, physical activity, daily energy intake, percentage of energy from protein, and BMI trajectory. The effect estimates and their 95% CIs represent the association between a 1-SD change in each metabolite and corresponding SD change in the CRM.

### Handling of missing covariates

All covariates except birth weight and length, breastfeeding duration and birth order had missing values. The range of percentages of missing covariates were 2–30% in males and 1–18% in females. Using SAS function ‘proc mi’, we created a single imputed dataset in one burn in iterations with the fully conditional method, linear regression for continuous variables (maternal BMI, gestational weight gain), logistic regression for ordinal categorical variables (smoking household), and discriminant function for nominal categorical variables (maternal education and maternal employment). Despite the single imputation’s limitation of not achieving uncertainty of missing data, we opted for this technique over the optimal multiple imputation because of the complexity of applying multiple imputation in longitudinal datasets^72^, the unstable results in longitudinal mixed-model analysis after multiple imputation^73^, and the generally low proportions of missingness in our data.

## Supplementary information

Supplementary results.

## Data Availability

The datasets generated during and/or analysed during the current study are available from the corresponding author on reasonable request and approval by the principal investigator.
