# Supplementary material for: Longitudinal relationship of amino acids and indole metabolites with long-term body mass index and cardiometabolic risk markers in young individuals
Source: Sci Rep. 2020 Apr 14;10:6399. doi: 10.1038/s41598-020-63313-z (PMC7156759; doi:10.1038/s41598-020-63313-z)
Supplement: Supplementary file 1 — Supplementary results. [file 41598_2020_63313_MOESM1_ESM.docx]

**Longitudinal relationship of amino acids and indole metabolites with long-term body mass index and cardiometabolic risk markers in young individuals**

Kolade Oluwagbemigun^1*^, Andrea Anesi^2^, Maria Ulaszewska^2^, Gerard Clarke^3,4,5^,

Ute Alexy^1^, Matthias Schmid^6^, Michael Roden^7,8,9^, Christian Herder^7,8,9^, Fulvio Mattivi^2,10^, Ute Nöthlings^1^

Affiliations:

^1^Nutritional Epidemiology, Department of Nutrition and Food Sciences, University of Bonn, Bonn, Germany

^2^Department of Food Quality and Nutrition, Research and Innovation Centre, Fondazione Edmund Mach (FEM), San Michele all’Adige, Italy

^3^APC Microbiome Ireland, University College Cork, Cork, Ireland

^4^INFANT Research Centre, University College Cork, Cork, Ireland

^5^Department of Psychiatry and Neurobehavioural Science, University College Cork, Cork, Ireland

^6^Department of Medical Biometry, Informatics and Epidemiology, University Hospital Bonn, University of Bonn, Bonn, Germany

^7^Division of Endocrinology and Diabetology, Medical Faculty, Heinrich Heine University Düsseldorf, Düsseldorf, Germany

^8^Institute for Clinical Diabetology, German Diabetes Center, Leibniz Center for Diabetes Research at Heinrich Heine University Düsseldorf, Düsseldorf, Germany^´^

^9^German Center for Diabetes Research (DZD), München-Neuherberg, Germany

^10^Department of Physics, University of Trento, San Michele all’Adige, Italy

*Corresponding author: Kolade Oluwagbemigun.

**Email:**  [koluwagb@uni-bonn.de](mailto:koluwagb@uni-bonn.de), ORCID: 0000-0002-9454-597.

**Keywords:** Amino acids; C-reactive protein; cardiometabolic risk markers; gut bacteria; indole metabolites; long-term body mass index trajectory groups; tryptophan

Supplemental Table 1a: Summary of the patterns of age (time) of urine sampling (metabolite measurement) in males (n=111)

Supplemental. Table 1b: Summary of the patterns of age (time) of urine sampling (metabolite measurement) in females (n=107)

Supplemental Table 2: Numbers represent the values of the corrected Akaike’s Information criterion of linear and quadratic time models

Supplemental Table 3: Mean difference in standard-deviation scores of indole-3-acetic acid between pairs of BMI trajectory groups in males, following additionally adjusted for its precursor

**Supplementary results**

**Suppl. Table 1a: Summary of the patterns of age (time) of urine sampling (metabolite measurement) in males (n=111)**

|  |  |  |  |  | **Participants’ age** | | |  |  |  |  |  |
| --- | --- | --- | --- | --- | --- | --- | --- | --- | --- | --- | --- | --- |
|  | **7** | **9** | **10** | **11** | **12** | **12.5** | **14** | **15** | **16** | **17** | **18** | **n (%)** |
| **Patterns** |  |  |  |  |  |  |  |  |  |  |  |  |
| **1** |  | X |  |  |  |  |  |  |  | X | X | 57 (51.4) |
| **2** |  | X |  |  |  |  |  |  | X | X |  | 10 (9) |
| **3** |  | X |  |  |  |  |  | X |  | X |  | 9 (8.1) |
| **4** |  | X |  |  |  |  |  | X | X |  |  | 6 (5.4) |
| **5** |  | X |  |  |  |  |  |  | X |  | X | 6 (5.4) |
| **6** |  |  | X |  |  |  |  |  |  | X | X | 4 (3.6) |
| **7** |  | X |  |  |  |  | X |  | X |  |  | 3 (2.7) |
| **8** | X |  |  |  |  |  |  |  |  | X | X | 2 (1.8) |
| **9** |  | X |  |  |  |  | X | X |  |  |  | 2 (1.8) |
| **10** | X |  |  |  |  |  |  |  | X | X |  | 1 (0.9) |
| **11** |  | X |  |  |  |  | X |  |  | X |  | 1 (0.9) |
| **12** |  | X |  |  |  |  | X |  |  |  | X | 1 (0.9) |
| **13** |  | X |  |  |  |  |  | X |  |  | X | 1 (0.9) |
| **14** |  |  | X |  |  |  | X | X |  |  |  | 1 (0.9) |
| **15** |  |  | X |  |  |  |  | . | X | X |  | 1 (0.9) |
| **16** |  |  |  | X |  |  |  | X |  | X |  | 1 (0.9) |
| **17** |  |  |  | X |  |  |  | X |  |  | X | 1 (0.9) |
| **18** |  |  |  | X |  |  |  |  | X | X |  | 1 (0.9) |
| **19** |  |  |  | X |  |  |  |  |  | X | X | 1 (0.9) |
| **20** |  | X |  |  | X |  |  |  | X |  |  | 1 (0.9) |
| **21** |  | X |  |  |  | X |  |  |  |  | X | 1 (0.9) |

“X” represents 24h urine sampling; n=count; %=percentage

**Suppl. Table 1b: Summary of the patterns of age (time) of urine sampling (metabolite measurement) in females (n=107)**

|  |  |  |  |  |  |  |  | **Age** |  |  |  |  |  |  |  |
| --- | --- | --- | --- | --- | --- | --- | --- | --- | --- | --- | --- | --- | --- | --- | --- |
|  | **7** | **8** | **8.5** | **9** | **9.5** | **10** | **11** | **12** | **13** | **14** | **15** | **16** | **17** | **18** | **n (%)** |
| **Patterns** |  |  |  |  |  |  |  |  |  |  |  |  |  |  |  |
| **1** |  |  |  | X |  |  |  |  |  |  |  |  | X | X | 64 (59.8) |
| **2** |  |  |  | X |  |  |  |  |  |  |  | X | X |  | 11 (10.3) |
| **3** |  |  |  | X |  |  |  |  |  |  |  | X |  | X | 8 (7.5) |
| **4** |  |  |  | X |  |  |  |  |  |  | X | X |  |  | 4 (3.7) |
| **5** |  |  |  | X |  |  |  |  |  |  | X |  | X |  | 3 (2.8) |
| **6** |  |  |  | X |  |  |  |  |  |  | X |  |  | X | 3 (2.8) |
| **7** | X |  |  |  |  |  |  |  |  |  | X |  | X |  | 1 (0.9) |
| **8** | X |  |  |  |  |  |  |  |  |  |  | X | X |  | 1 (0.9) |
| **9** | X |  |  |  |  |  |  |  |  |  |  |  | X | X | 1 (0.9) |
| **10** |  |  |  | X |  |  | X |  |  | . | X |  |  |  | 1 (0.9) |
| **11** |  |  |  | X |  |  |  |  |  | X | X |  |  |  | 1 (0.9) |
| **12** |  |  |  | X |  |  |  |  |  | X |  |  | X |  | 1 (0.9) |
| **13** |  |  |  | X |  |  |  |  |  | X |  |  |  | X | 1 (0.9) |
| **14** |  |  |  |  |  |  | X |  |  | . |  |  | X | X | 1 (0.9) |
| **15** |  | X |  |  |  |  |  |  |  |  |  |  | X | X | 1 (0.9) |
| **16** |  |  | X |  |  |  |  |  |  |  |  |  | X | X | 1 (0.9) |
| **17** |  |  |  | X |  |  |  | X |  |  | X |  |  |  | 1 (0.9) |
| **18** |  |  |  | X |  |  |  |  | X |  | X |  |  |  | 1 (0.9) |
| **19** |  |  |  |  | X |  |  |  |  |  |  |  | X | X | 1 (0.9) |
| **20** |  |  |  |  |  | X |  |  |  |  |  | X | X |  | 1 (0.9) |

“X” represents 24h urine sampling; n=count; %=percentage

**Suppl. Table 2: Numbers represent the values of the corrected Akaike’s Information criterion of linear and quadratic time models**

|  | **MALES** |  | **FEMALES** |  |
| --- | --- | --- | --- | --- |
| Metabolites* | Linear | Quadratic | Linear | Quadratic |
| GABA | 806.4 | 810.3 | 788.2 | 792.8 |
| Met | 932.4 | 935.5 | 903.2 | 907.0 |
| Val | 903.3 | 906.7 | 912.9 | 916.5 |
| Leu | 910.1 | 914.1 | 913.0 | 916.3 |
| Ile | 910.2 | 913.6 | 913.0 | 913.8 |
| PA | 936.8 | 939.3 | 912.8 | 917.2 |
| QA | 905.6 | 908.8 | 912.6 | 917.1 |
| 5-HT | 926.2 | 928.6 | 912.8 | 917.0 |
| DA | 947.6 | 948.6 | 903.0 | 906.9 |
| Tyr | 882.7 | 886.5 | 914.0 | 916.5 |
| Phe | 902.7 | 905.9 | 913.0 | 916.4 |
| Tyra | 889.4 | 893.4 | 884.8 | 888.8 |
| 3-MeTyra | 896.6 | 900.6 | 912.8 | 916.8 |
| HVA | 910.9 | 915.4 | 913.0 | 917.3 |
| KA | 931.0 | 935.1 | 913.0 | 916.9 |
| 5-OH-IAA | 936.0 | 939.2 | 913.5 | 917.8 |
| Trp | 898.8 | 901.8 | 913.0 | 917.0 |
| **Kyn** | **906.3** | **904.4** | 913.0 | 916.6 |
| XA | 938.0 | 942.5 | 901.9 | 906.0 |
| IACT | 936.1 | 940.5 | 899.4 | 903.8 |
| IAA | 937.4 | 941.8 | 879.9 | 884.3 |
| ILA | 911.9 | 916.4 | 887.0 | 890.8 |
| IPA | 912.4 | 916.6 | 883.3 | 887.5 |
| IALD | 944.9 | 949.2 | 904.5 | 908.8 |
| ICA | 931.2 | 934.9 | 912.8 | 917.2 |
| AA | 934.6 | 939.0 | 912.8 | 915.9 |
| 3-OH-AA | 926.9 | 930.3 | 911.4 | 915.6 |
| Try | 881.1 | 881.8 | 869.2 | 872.7 |
| 5-Me-IAA | 913.9 | 916.9 | 913.1 | 916.6 |
| TrpME | 948.8 | 952.6 | 897.5 | 900.8 |
| 5-OH-Trp | 929.9 | 932.2 | 912.5 | 916.6 |
| 3-OH-Kyn | 897.6 | 901.5 | 885.1 | 888.0 |
| **KTR** | **839.3** | **834.9** | 845.7 | 849.5 |

GABA=Gamma-aminobutyric acid; Met=Methionine; Val=Valine; Leu=Leucine; Ile=Isoleucine; PA=Picolinic acid; QA=Quinolinic acid; 5-HT=Serotonin; DA=Dopamine; Tyr=Tyrosine; Phe=Phenylalanine; Tyra=Tyramine; 3-Me-Tyra=3-methoxy-p-tyramine; HVA=Homovanillic acid; KA=Kynurenic acid; 5-OH-IAA=5-hydroxyindole-3-acetic acid; Trp=Tryptophan; Kyn=Kynurenine; XA=Xanthurenic acid; IACT=Indole-3-acetamide; IAA=Indole-3-acetic acid; ILA=Indole-3-lactic acid; IPA=Indole-3-propionic acid; IALD=Indole-3-carboxaldehyde; ICA=Indole-3-carboxylic acid; AA=Anthranilic acid; 3-OH-AA=3-hydroxyanthranilic acid; Try=Tryptamine; 5-Me-IAA=5-Methoxyindole-acetic acid; TrpME=Tryptophan methyl ester; 5-OH-Trp=5-hydroxy-tryptophan, 3-OH-Kyn=3-hydroxykynurenine. KTR= Kynurenine to Tryptophan ratio

The bolded metabolites indicate that quadratic time model fit better than linear time model

*Metabolites and time were normalized before statistical analyses

**Suppl. Table 3: Mean difference in standard-deviation scores of indole-3-acetic acid between pairs of BMI trajectory groups in males following additional adjustment for tryptophan**

| **Metabolites** | Difference in standardized mean (95% Confidence interval) | P-value* |
| --- | --- | --- |
| **Indole-3-acetic acid** |  |  |
| Overweight vs. High-normal weight | -0.558 (-0.978; -0.138) | 0.001 |
| Overweight vs. Mid-normal weight | -0.721 (-1.144; -0.299) | <0.001 |
| Overweight vs. Low-normal weight | -0.557 (-1.020; -0.095) | 0.002 |
| High-normal weight vs. Mid-normal weight | -0.163 (-0.466; 0.139) | 0.165 |
| High-normal weight vs. Low-normal weight | 0.001 (-0.346; 0.347) | 0.996 |
| Mid-normal weight vs. Low-normal weight | 0.164 (-0.182; 0.510) | 0.222 |

Model adjusted for time, birth weight and length, maternal BMI, maternal pregnancy weight gain, breastfeeding duration, birth order, maternal education, and maternal employment, smoking in household, physical activity, daily energy intake, percentage of energy from protein, and two-way interactions of physical activity, daily energy intake, and percentage of energy from protein with BMI trajectory, and tryptophan.

*Simulated adjusted *p*-value
